# Variability in the utilization of preventive dental services among the underserved population of Indiana

**DOI:** 10.1371/journal.pone.0337471

**Published:** 2025-11-24

**Authors:** Anubhuti Shukla, Bhavya Vaishnavi Amrutham, Sheetal Manchanda

**Affiliations:** 1 Associate Professor, Department of Oral Health Sciences, Maurice H. Kornberg School of Dentistry, Temple University, Philadelphia, Pennsylvania, United States of America; 2 D.M.D. Candidate, Nova Southeastern University College of Dental Medicine, Clearwater, Florida, United States of America; 3 D.D.S. Candidate, Indiana University School of Dentistry, Indianapolis, Indiana, United States of America; Shahid Beheshti University of Medical Sciences School of Dentistry, IRAN, ISLAMIC REPUBLIC OF

## Abstract

**Objectives:**

To explore the variability of utilization patterns of preventive dental services among diverse demographic groups in the state of Indiana.

**Methods:**

This study utilizes a retrospective data analysis approach to examine the preventive dental services utilization among patients treated by senior-year dental students (D4 students) during their community-based clinical rotations at Federally Qualified Health Centers and private practices, affiliated with Indiana University School of Dentistry. The data were extracted from daily patient reports submitted by D4 students for every patient they examined during their rotations, via RedCap. Descriptive statistics and generalized linear modeling using main effects were performed to identify the utilization of different preventive services across urbanization stratification and demographic variables among the included participants.

**Results:**

There was a statistically significant (p < 0.001) distribution of all dental preventive services according to demographic characteristics, viz. age, ethnicity, location (rural or urban), primary language spoken, and insurance status. There were 30.39 times higher odds of utilizing preventive dental services in a rural zip code compared to an urban zip code. Individuals with multicultural ethnicities (OR= 0.91, 95% CI: 0.54, 1.53) were less likely to utilize preventive dental services than the “Other” category, although the value was not significant (p = 0.72). Also, the younger age group (less than 17 years) was more likely to utilize preventive dental services than the older age group (p < 0.001).

**Conclusions:**

Inequities in the utilization of preventive dental services were identified among the underserved population of Indiana, according to demographic characteristics.

## Introduction

Regular dental visits and consistent care positively influence long-term oral health outcomes and rely on an individual’s understanding of the importance of preventive measures [1]. However, the high prevalence of unmet dental needs among the adult population of the US highlights the need to explore the underlying contributing factors [2,3]. In addition, a decreasing trend of dental service utilization among adults and young children in the US has been reported [4,5]. Moreover, preventive dental care, such as topical fluoride applications and dental sealants, has been found to significantly correlate with reduced non-preventive visits and dental expenditures, suggesting that preventive care can lead to overall cost savings and potentially improved oral health outcomes [6]. Furthermore, a study done across six states of the US reported a substantial annual cost savings of approximately $1.1 million in Mississippi to $12.9 million in Texas, from implementing the preventive services at a 10% utilization rate [7]. Also, Medicaid-enrolled children who did not receive preventive dental services have been found to require treatment for dental issues at an earlier age than their counterparts who had access to preventive dental care [8].

Although there have been notable advancements in oral health, persistent disparities remain, largely influenced by socio-demographic determinants. The rural-urban differences in the utilization rates of dental services have been observed among US adults [9]. The geographic disparities in the utilization of various dental services were reported with lower rates of preventive dental services and higher rates of tooth extraction [10]. Similarly, individuals from lower socio-economic backgrounds had less frequent dental visits and reduced access to preventive care, thus contributing to a higher incidence of oral diseases [11,12]. These challenges are more pronounced among American children from minority and economically disadvantaged backgrounds [13,14]. Moreover, these disparities have been found to be associated with the presence of linguistic and cultural differences [15].

While socio-demographic factors have been extensively studied concerning general healthcare access, there is a lack of focused research examining how these factors influence preventive dental care, particularly within the state of Indiana, United States. The lack of comprehensive data specific to underserved communities highlights the need for more detailed studies to understand the socioeconomic barriers affecting the utilization of preventive dental services. This study aims to address this gap by exploring the variability of utilization patterns of preventive dental services among diverse demographic groups in the state of Indiana. This study might help uncover actionable insights that could inform policy interventions aimed at reducing disparities and improving access to preventive dental services and thus oral health outcomes.

## Methodology

This study utilizes a retrospective data analysis approach to examine the preventive dental services utilization among patients treated by senior-year dental students (D4 students) during their community-based clinical rotations at Federally Qualified Health Centers (FQHCs) and private practices, affiliated with Indiana University School of Dentistry (IUSD). The data were extracted from daily patient reports submitted by D4 students for every patient they examined during their rotations, *via* RedCap, an electronic data capture system, and Heto [16] (Cabaana Inc., Michigan, USA), a cloud-based student scheduling software.

The data utilized in this study consists of patient demographic information and treatment details from October 2019 to May 2024 and was accessed on 15-11-2024. The patient demographic variables included age (categorized into nine groups: 0–6 years, 7–12 years, 13–17 years, 18–24 years, 25–34 years, 35–44 years, 45–54 years, 55–64 years, 65 or older), Race/Ethnicity (White, Hispanic or Latino, Black or African American, Multicultural, and Other), Primary Language (English, Spanish, or Other), Insurance type [Medicaid, Medicare, Private Insurance, Uninsured, and others including Children’s health Insurance Program (CHIP), Healthy Indiana Plan (HIP), and Federal Sliding Fee Scale].

Provider’s location was geocoded to the zip code level (with the location of the dental practices classified into urban and rural, based on the practice’s corresponding postal codes) using zip code tabulation areas (ZCTAs) produced by Health Resources & Services Administration (HRSA) by the US Department of Health & Human Services [17].

Clinical data were recorded using Current Dental Terminology (CDT) Codes to document the treatments performed on the patients. For this study, only preventive treatments were considered, identified by the following CDT codes [18]: Prophylaxis – Adult (D1110), Prophylaxis– Child (D1120), Topical Fluoride application including Varnish (D1206) and excluding Varnish (D1208), Oral Hygiene Instructions (D1330), Sealant – Per Tooth (D1351), Preventive Resin Restoration in a Moderate to High Caries Risk Patient – Permanent Tooth (D1352), Silver Diamine Fluoride (SDF) (D1354).

The criteria for inclusion in the study were patients receiving the above-specified preventive dental services as outlined by the above CDT codes. Any records lacking specific information on the services performed or falling outside the scope of preventive care were excluded.

This study was conducted in accordance with the guidelines and regulations set forth by the Institutional Review Board (IRB) of Indiana University. The protocol number for this study is #22902, and it was determined to be exempt from full IRB review. Data used in this retrospective observational study were completely anonymized before being accessed by the study team. The study data were accessed in November 2024.

### Statistical analysis

Data were analyzed using the Statistical Package for the Social Sciences (SPSS) version 29 for Windows (IBM Corp. Released 2023. IBM SPSS Statistics for Windows, Version 29.0.2.0 Armonk, NY: IBM Corp). Descriptive statistics were performed to identify the utilization of different preventive services across urbanization stratification and demographic variables of the included participants, using the Pearson chi-square test. Generalized Linear Modeling (GLM) using main effects was used to evaluate the effect estimates since the dependent variable (preventive dental service utilized) did not follow a normal distribution. The predictor variables imputed into the model were urbanization, ethnicity, age, primary language spoken, and dental insurance status. All statistical analyses were performed at an alpha of 0.05 with 95% CI (confidence intervals).

## Results

**Table 1** shows the demographic characteristics of the 1409 sample population as per the preventive dental services they received. Child Prophylaxis was the most common preventive dental service administered (n = 376; 26.69%), followed by adult prophylaxis (n = 301; 21.36%). The utilization of all types of preventive dental services varied significantly across demographic groups. Statistically significant differences (p < 0.001) were observed based on age, ethnicity, rural versus urban location, primary language spoken, and insurance status. As can be seen, nearly 1/5^th^ of the adult prophylaxis was performed in the 35–44 age group, whereas about half of the child prophylaxis was done in children less than 6 years of age. Moreover, all the preventive dental services were utilized mostly by participants of Caucasian descent or people of Hispanic origin. As expected, most of the other preventive services, like sealants and fluorides, were more commonly performed in the younger age groups. Surprisingly, the majority of the preventive dental services were provided in the locations that were classified as urban, as per the zip codes. As presented in **Table 1**, Medicaid was the most common insurance status used for all dental services, except for adult prophylaxis, for which the majority of the participants were uninsured. Furthermore, English was the primary spoken language for all the preventive dental services utilized.

**Table 1:** Utilization of preventive dental services according to the demographic characteristics.

	Adult prophylaxis	Child prophylaxis	Topical fluoride	Oral hygiene instructions	Sealants	Preventive resin restorations	Silver diamine fluoride	p-value
**Age**								<0.001*
≤ 6 years	1 (0.33%)	191 (50.80%)	111 (46.64%)	23 (19.49%)	20 (7.69%)	1 (1.85%)	30 (48.39%)	
7-12 years	7 (2.33%)	157 (41.76%)	57 (23.95%)	23 (19.49%)	134 (51.54%)	3 (5.56%)	14 (22.58%)	
13-17 years	34 (11.30%)	20 (5.32%)	31 (13.02%)	10 (8.47%)	94 (36.15%)	6 (11.11%)	2 (3.23%)	
18-24 years	43 (14.29%)	5 (1.33%)	7 (2.94%)	8 (6.78%)	3 (1.15%)	9 (16.67%)	0 (0.00%)	
25-34 years	58 (19.27%)	2 (0.53%)	12 (5.04%)	13 (11.02%)	5 (1.92%)	11 (20.37%)	1 (1.61%)	
35-44 years	61 (20.27%)	0 (0.00%)	4 (1.68%)	11 (9.32%)	1 (0.38%)	11 (20.37%)	10 (16.13%)	
45-54 years	41 (13.62%)	0 (0.00%)	6 (2.52%)	13 (11.02%)	2 (0.77%)	7 (12.96%)	0 (0.00%)	
55-64 years	32 (10.63%)	0 (0.00%)	7 (2.94%)	14 (11.86%)	1 (0.38%)	5 (9.26%)	3 (4.84%)	
65 years & above	24 (7.97%)	1 (0.27%)	3 (1.26%)	3 (2.54%)	0 (0.00%)	1 (1.85%)	2 (3.23%)	
**Ethnicity**								<0.001*
White	135 (51.14%	105 (30.00%)	69 (30.713%)	38 (37.62%)	83 (34.30%)	10 (23.81%)	20 (37.74%)	
Hispanics/Latino	87 (32.95%)	128 (36.57%)	109 (47.60%)	38 (37.62%)	85 (35.12%)	19 (45.24%)	18 (33.96%)	
Black/African American	28 (10.61%)	70 (20.00%)	35 (15.28%)	16 (15.84%)	59 (24.38%)	8 (19.05%)	12 (22.64%)	
Multicultural	10 (3.79%)	14 (4.00%)	7 (3.06%)	6 (5.94%)	0 (0.00%)	2 (4.76%)	0 (0.00%)	
Others	4 (1.52%)	33 (9.43%)	9 (3.93%)	3 (2.97%)	15 (6.20%)	3 (7.14%)	3 (5.66%)	
**Location**								<0.001*
Rural	30 (9.97%)	16 (4.26%)	17 (7.14%)	5 (4.24%)	47 (18.08%)	1 (1.85%)	5 (8.06%)	
Urban	270 (90.00%)	360 (95.74%)	221 (92.86%)	113 (95.76%)	213 (81.92%)	53 (98.15%)	57 (91.94%)	
**Primary language spoken**								<0.001*
English	209 (69.44%)	265 (70.48%)	148 (62.18%)	79 (66.95%)	209 (80.38%)	28 (51.85%)	47 (75.81%)	
Spanish	79 (26.25%)	94 (25.00%)	79 (33.19%)	34 (28.81%)	45 (17.31%)	19 (35.19%)	12 (19.35%)	
Others	13 (4.32%)	17 (4.52%)	11 (4.62%)	5 (4.24%)	6 (2.31%)	7 (12.96%)	3 (4.84%)	
**Insurance status**								<0.001*
Medicaid	102 (33.89%)	193 (51.33%	124 (52.10%)	51 (43.22%)	147 (56.54%)	22 (40.74%)	29 (46.77%)	
Medicare	8 (2.66%)	3 (0.80%)	1 (0.42%)	2 (1.69%)	3 (1.15%)	1 (1.85%)	0 (0.00%)	
Private Insurance	36 (11.96%)	64 (17.02%)	34 (14.29%)	10 (8.47%)	38 (14.62%)	2 (3.70%)	10 (16.13%)	
Uninsured	122 (40.53%)	67 (17.82%)	31 (13.03%)	32 (27.12%)	37 (14.23%)	19 (35.19%)	15 (24.19%)	
Other insurance	33 (10.96%)	49 (13.03%)	48 (20.17%)	23 (19.49%)	35 (13.46%)	10 (18.52%)	8 (12.90%)	
**Total**	301 (21.36%)	376 (26.69%)	238 (16.89%)	118 (8.37%)	260 (18.23%)	54 (3.83%)	62 (4.40%)	

*Chi-square test.

The Omnibus test to evaluate the fit of the GLM model, with the predictor variables to the intercept-only model, was found to be significant (p < 0.001), indicating that our model was better than the intercept-only model. As shown in **Table 2**, except for the primary language spoken (p = 0.143), all other predictor variables were significantly associated with preventive dental service utilization. There were 30.39 times higher odds of utilizing preventive dental services in a rural zip code compared to an urban zip code. Individuals with multicultural ethnicities (OR= 0.91, 95% CI: 0.54, 1.53) were less likely to utilize preventive dental services than the “Other” category, although the value was not significant (p = 0.72). Also, the younger age group (less than 17 years) was more likely to utilize preventive dental services than the older age group (p < 0.001). There were almost two times higher odds of utilizing preventive dental service by patients who were privately insured (OR= 2.21, 95% CI: 1.68, 2.91) compared to the reference group patients (Other insurance) (p < 0.001). Similarly, patients who were on Medicaid had nearly 1.27 times higher odds of utilizing preventive dental services compared to the people who had “Other” insurance (95% CI: 1.03, 1.57).

**Table 2:** Regression model using Generalized Linear equation (age, ethnicity, primary language, location, and insurance status) on preventive dental services utilization.

	Odds Ratio [Exp (B)]	95% CI	p-value	Overall p-value (tests of model effects) *
**Age**				**<0.001**
≤ 6 years	48.99	32.58, 73.67	**<0.001**	
7 ─ 12 years	25.97	17.50, 38.55	**<0.001**	
13 ─ 17 years	8.11	5.42, 12.13	**<0.001**	
18 ─ 24 years	1.74	1.13, 2.69	**0.01**	
25 ─ 34 years	1.07	0.71, 1.62	0.75	
35 ─ 44 years	1.03	0.68, 1.57	0.88	
45 ─ 54 years	0.80	0.51, 1.23	0.31	
55 ─ 64 years	0.94	0.61, 1.47	0.79	
≥ 65 years	1	─	─	
**Ethnicity**				**<0.001**
White	1.01	0.70, 1.47	0.96	
Hispanic or Latino	1.36	0.92, 2.02	0.12	
Black or African American	1.42	0.96, 2.09	0.08	
Multicultural	0.91	0.54, 1.53	0.72	
Others	1	─	─	
**Location**				**<0.001**
Rural	30.39	19.04, 48.52	**<0.001**	
Urban	1	─	─	
**Primary language spoken**				0.143
English	1.46	1.00, 2.13	**0.05**	
Spanish	1.43	0.94, 2.19	**0.09**	
Others	1	─	─	
**Insurance status**				**<0.001**
Medicaid	1.27	1.03, 1.57	**0.02**	
Medicare	0.62	0.35, 1.09	0.10	
Private insurance	2.21	1.68, 2.91	**<.001**	
Uninsured	1.03	0.82, 1.29	0.80	
Other insurance	1	─	─	

*Generalized Linear Modeling.

## Discussion

The findings of the study provide an update on the utilization of preventive dental services for the study period between the years 2019–2024, among the underserved population of Indiana. To the best of our knowledge, this is the first study conducted in the state of Indiana, evaluating the preventive dental services utilization pattern, categorized into different levels of urbanization according to the dental provider’s zip codes.

The present study corroborates the results of previous studies that patients residing in urban areas receive more preventive dental services than those living in rural communities [19,20]. However, on multivariate analysis, there were almost 30 times higher odds of dental service utilization in rural areas than in urban areas. Thus, although there is a higher absolute dental service utilization in urban areas, people residing in rural areas are more likely to utilize preventive services. This could also be due to a higher number of services in urban areas because of the larger population or higher access, rather than a lower utilization rate in rural areas because of the lack of options and lesser access. Moreover, the foremost use of professional dental cleaning among all preventive services was found significantly higher among those visiting dentists located in urban areas. An earlier study confirmed this rural-urban disparity in the utilization rates of preventive services [21]. Another aspect to consider is that preventive dental services for adults (prophylaxis and oral hygiene instructions) were least received by patients older than 65 years of age, highlighting a glaring gap faced by the older population. This might be attributed to the barriers commonly faced by geriatric patients that might significantly decrease their access to preventive dental visits and thus, a lesser number of preventive services [22].

According to the US Preventive Services Task Force, topical fluoride application is a recommended caries preventive strategy for children. The present study results demonstrated the implementation of the topical fluoride preventive measure in a substantial proportion of children. In addition, the use of silver diammine fluoride was most common in the younger age group, which is expected as SDF is often applied to postpone more complex treatments that may require general anesthesia (GA) or sedation in uncooperative children or to arrest dental caries in patients waiting for GA [23,24].

Oral health strategies developed by the US Department of Health and Human Services, like Healthy People 2020 and the Affordable Care Act, emphasize the importance of preventing oral diseases and recognize barriers like financial and lack of dental insurance coverage [25]. The present study demonstrated the predominant percentage of participants using Medicaid for dental insurance coverage. Although insurance coverage might be one of the factors that affect the utilization of dental services, the rates of dental care utilization have been found to remain low even in states that have an expansion of Medicaid coverage benefits [26,27]. The findings of the current study highlight that a substantial number of participants lacked insurance for receiving preventive services, a finding that aligns with a national survey [28], that pinpointed lower utilization of dental sealants among uninsured US children. However, the implementation of the Affordable Care Act in the US has shown a reduction in the cost barrier for dental care from 15.0% to 11.7%, with a decline in the number of uninsured adult [29,30]. A previous report identified the persistent inequities in dental care and recommended further strengthening of these policies [31].

Our findings concur with the earlier reports [32–34], confirming greater utilization of dental services by English-speaking individuals as compared to those speaking any other language. This disparity might be attributed to the language barriers encountered during the dental visits by non-English-speaking patients, which preclude them from using these services. Thus, our study findings highlight the prevalence of such disparities and call for attention to eliminate or reduce them through cultural sensitivities, personalized care, and effective communication strategies like interpreter-mediated dental care [35].One of the limitations of this study is its retrospective cross-sectional design, which makes it difficult to establish causation between the variables and the outcome. Additionally, a significant part of the data (2020−2022) was collected during the COVID-19 pandemic, which could have impacted the utilization rates. Additionally, most of the dental treatment during this time centered around minimally invasive procedures like SDF application instead of traditional cavity preparations to combat aerosol production, which might affect the generalizability in a non-pandemic context [36,37]. Moreover, the current study lacked data on participants visiting other dentists or healthcare providers for any preventive dental services. It is crucial to recognize that the American Academy of Pediatrics recommends pediatricians’ role in the US for supporting children’s oral health, by conducting caries risk assessment, fluoride varnish application, and oral health education for families [38].

On the contrary, the present study has its strength in that it conducted a multivariate analysis to evaluate the disparities among the underserved population of Indiana state for preventive dental services, to help uncover the potential barriers, and to inform policy decisions. Moreover, as the healthcare system varies across different states of the US, this state-level presentation of our study findings pinpoints the *“no one-size-fits-all”* approach for tailoring policies and interventions that best cater to the Indiana population and understand the identified concerns. For instance, the state-specific Medicaid programs could consider these disparities while formulating dental care decisions.

In conclusion, disparities in the utilization of preventive dental services were identified among the underserved population of Indiana, according to demographic characteristics, *viz.,* age, ethnicity, location (rural or urban), primary language spoken, and insurance status. The study findings describe the disparities associated with race/ethnicity and insurance status, thus recommending that policymakers consider them while making dental care actions and decisions.

## Supporting information

S1 File 2019-2024 reg. (XLSX)

S2 File Analysis code book. (DOCX)
