# Supplementary material for: Variability in the utilization of preventive dental services among the underserved population of Indiana
Source: PLoS One. 2025 Nov 24;20(11):e0337471. doi: 10.1371/journal.pone.0337471 (PMC12643307; doi:10.1371/journal.pone.0337471)
Supplement: S2 File — (DOCX) [file pone.0337471.s002.docx]

**=IF(M2=1,1,IF(N2=1,2,IF(O2=1,3,IF(P2=1,4,IF(Q2=1,5,IF(R2=1,6,IF(S2=1,7,IF(T2=1,8,IF(U2=1,9,IF(V2=1,10,IF(W2=1,11,IF(X2=1,12,IF(Y2=1,13,IF(Z2=1,14,IF(AA2=1,15,"0")))))))))))))))**

**=IF(AB2=1, "D1110",**

**IF(AB2=2, "D1120",**

**IF(AB2=3, "D1206",**

**IF(AB2=4, "D1208",**

**IF(AB2=5, "D1330",**

**IF(AB2=6, "D1351",**

**IF(AB2=7, "D1352",**

**IF(AB2=8, "D1354",**

**IF(AB2=9, "D1510",**

**IF(AB2=10, "D1515",**

**IF(AB2=11, "D1520",**

**IF(AB2=12, "D1525",**

**IF(AB2=13, "D1550",**

**IF(AB2=14, "D1575",**

**IF(AB2=15, "D1555", "")))))))))))))))**

**=IF(AND(AB2>=1, AB2<=15), 1, "0")


Age : 2019 -2024**

0-6 years – 1

7-12 years -2

13-17 years – 3

18-24 years -4

25-34 years – 5

35-44 years -6

45-54 years -7

55-64 years – 8

65-74 years – 9

75 years or older -10

**Ethnicity 2019, 2020**

White: 1

Hispanic or Latino: 2

Black or African American – 3

Multicultural – 4

Other: 5

**Ethnicity : 2021, 2022, 2023**

White : 1

Hispanic or Latino : 2

Black or African American – 3

Multicultural – 4

Other race: 5 (American Indian/ Alaskan native ; Native Hawaiian / pacific islander, asian)

**2024 :**

White : 1

Hispanic or Latino: 2

Black or African American – 3

Multicultural – 4

Other race: 5 (Asian; American Indian/ Alaskan native ; Native Hawaiian / pacific islander; Middle easterners/ North Africans ; Two/ more races; Other races)

**Primary language : 2019 - 2024**

English :1

Spanish: 2

Other: 3

**Insurance status 2019 , 2020**

Medicaid: 1

Medicare: 2

Private insurance: 3

Uninsured: 4

Other: 5 (CHIP)

**Insurance status 2021**

Medicaid: 1

Medicare: 2

Private insurance: 3

Uninsured: 4

Other: 5 (Federal sliding Fee scale, Healthy Indiana plan)

**Insurance status 2024**

Medicaid: 1

Medicare: 2

Private insurance: 3

Uninsured: 4

Other: 5 (HIP (healthy Indiana plan, Federal sliding Fee scale)

**Preventive 2019 - 2024**

D1110-Prophylaxis – adult :1

D1120-Prophylaxis – child : 2

D1206-Topical Fluoride varnish = 3

D1208-Topical application of fluoride - excluding varnish : 4

D1330-Oral Hygiene Instructions :5

D1351-Sealant - per tooth :6

D1352-Preventive resin restoration in a moderate to high caries risk patient - permanent tooth :7

D1354-SDF :8

D1510-Space maintainer - fixed – unilateral :9

D1515-Space maintainer - fixed – bilateral : 10

D1520-Space maintainer - removable – unilateral :11

D1525-Space maintainer - removable – bilateral :12

D1550-Re-cement or re-bond space maintainer :13

D1575-Distal shoe space maintainer - fixed/unilateral :14

D1555-Removal of fixed space maintainer : 15
